# Management of nipple–areolar necrosis in a patient with Klippel-Trenaunay Syndrome

**DOI:** 10.1093/jscr/rjaf776

**Published:** 2025-09-28

**Authors:** Aulon Jerliu, Rebecca Hudon, Virginia Byars, Jillian Fortier

**Affiliations:** Department of Plastic and Reconstructive Surgery, University of Connecticut School of Medicine, 263 Farmington Ave, Farmington, CT 06030, United States; Department of Surgery, University of Connecticut School of Medicine, 263 Farmington Ave, Farmington, CT 06030, United States; Department of Plastic and Reconstructive Surgery, University of Connecticut School of Medicine, 263 Farmington Ave, Farmington, CT 06030, United States; Department of Surgery, University of Connecticut School of Medicine, 263 Farmington Ave, Farmington, CT 06030, United States; Department of Plastic and Reconstructive Surgery, University of Connecticut School of Medicine, 263 Farmington Ave, Farmington, CT 06030, United States; Department of Surgery, University of Connecticut School of Medicine, 263 Farmington Ave, Farmington, CT 06030, United States; Department of Plastic and Reconstructive Surgery, University of Connecticut School of Medicine, 263 Farmington Ave, Farmington, CT 06030, United States; Department of Surgery, University of Connecticut School of Medicine, 263 Farmington Ave, Farmington, CT 06030, United States

**Keywords:** Klippel-Trenaunay Syndrome, nipple–areolar complex necrosis, capillary malformations, DMSO

## Abstract

We present a case of a 49-year-old female with Klippel-Trenaunay Syndrome who developed partial thickness nipple–areolar complex (NAC) necrosis following explantation of breast implants with concurrent mastopexy. Notably, she had received pulse dye laser therapy to her left breast 2 years prior for treatment of a congenital capillary vascular malformations. Initial management included the use of nitropaste topically to ischemic left breast nipple areola complex, however, due to intolerance patient was switched to topical dimethyl sulfoxide, yielding improvement. This case highlights potential microvascular compromise in patient with capillary malformations of the breast undergoing breast surgery and suggests a successful application of dimethyl sulfoxide in treating NAC ischemic complications. The patient declined consent for clinical photography; therefore, we provide detailed narrative descriptions and anatomical measurements. At 12-week follow-up, the NAC had re-epithelialized completely with preserved projection and excellent symmetry. To our knowledge, this is the first reported case of this kind.

## Introduction

Breast implant removal combined with mastopexy is a frequently performed for women addressing complications from prior augmentation or desiring smaller breast volume and contour. Although rare, nipple–areolar complex (NAC) necrosis is a serious complication. Prior laser treatment may further impair perfusion and healing.

Klippel-Trenaunay Syndrome (KTS) is a rare congenital vascular disorder characterized by a triad of capillary venous malformations (CVM), venous anomalies, and limb overgrowth [1]. Involvement of the breast is less common but can manifest as extensive CVM. CVMs are often treated with pulse dye laser (PDL), a modality that may alter vascular dynamics [2–4].

PDL targets hemoglobin to induce thermal injury to abnormal vessels, a principle known as selective photothermolysis. While effective for lightening lesions, repeated treatments may result in dermal fibrosis, vessel wall thickening, and reduced capillary density, thus limiting tissue perfusion in treated skin. Histology shows chronic inflammation and endothelial loss after PDL [5, 6].

Recent investigations have explored pharmacologic strategies to improve skin flap viability in ischemic tissue. Dimethyl sulfoxide (DMSO) has emerged as an adjunctive treatment. DMSO’s mechanism of action includes scavenging hydroxyl radicals, improving microcirculation, and modulating inflammatory cytokines. It has been used in frostbite, radiation injury, flap salvage, and experimental wound models with encouraging results [7–9].

## Case presentation

**Figure 1:**
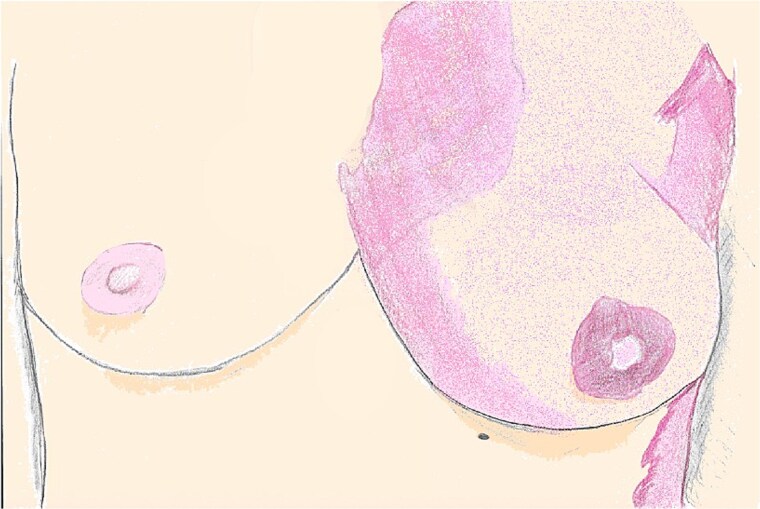
An illustration of bilateral breasts seen in the preoperative setting, with the CVM distribution seen across the left chest, and its absence across the right chest wall.

A 49-year-old female with a history of KTS involving the left chest wall presented for bilateral breast implant removal and mastopexy. Her medical history included treatment of a left breast CVMs with multiple rounds of PDL 2 years prior to surgery. On physical exam, the CVMs appeared as a violaceous, blanchable plaque extending across the upper outer quadrant of the left breast and lateral chest wall, consistent with a port-wine stain (Fig. 1).

**Figure 2:**
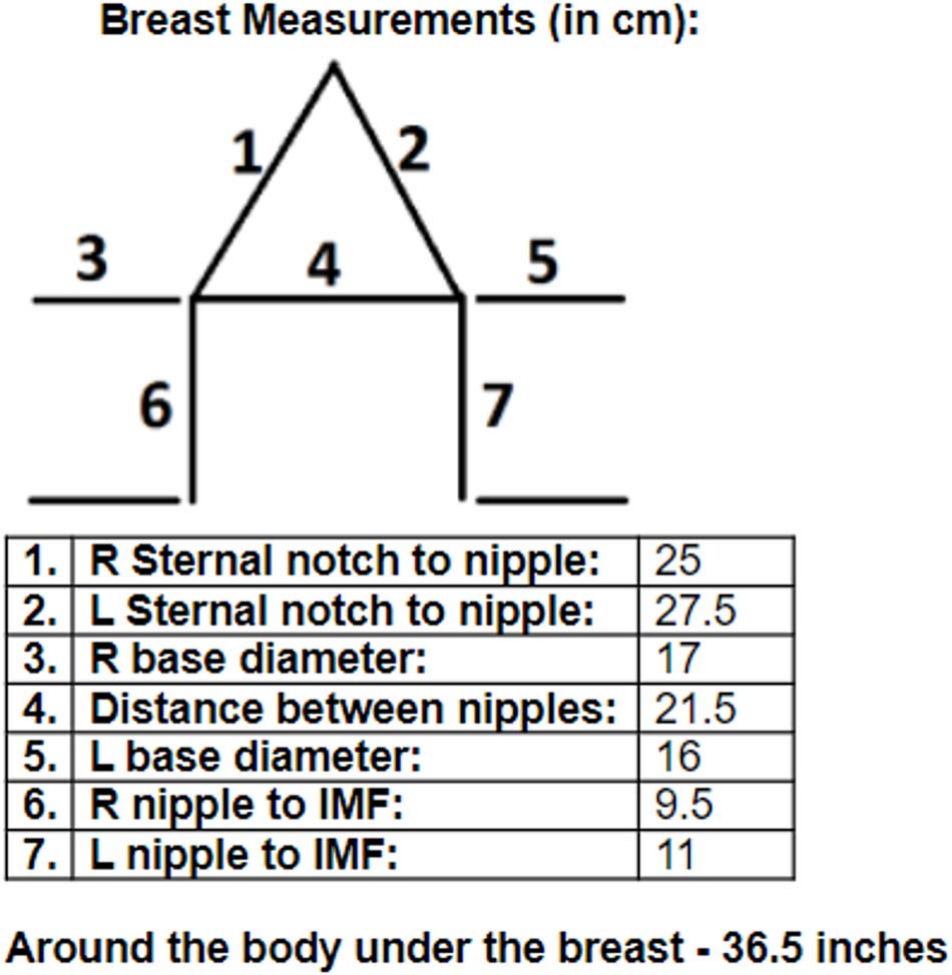
Preoperative breast measurements.

A Wise pattern mastopexy was planned using preoperative breast measurements (Fig. 2). Preoperative markings included a 9 cm vertical limb (extending from intended inframammary fold (IMF) to top of NAC) and a 38 mm areolar diameter, with planned nipple elevation 2 cm above the IMF. Intact McGhan 240 cc saline implants were removed bilaterally without capsulectomy due to soft and asymptomatic capsules. The areolar and lower pole skin was de-epithelialized, with limited medial, superior, and lateral undermining to preserve perforators to the central mound of breast tissue, thereby creating a broad-based central parenchymal pedicle. Undermining was performed only to the extent that allowed for the skin envelope to be re-draped over the breast mound and did not extend all the way down to the pectoralis major fascia. While no overt signs of ischemia were seen intraoperatively, perfusion was difficult to assess visually due to background violaceous discoloration from CVM. Skin excision totaled 17 g (right) and 22 g (left).

On postoperative Day 8, patient was seen by surgeon and noted to have dusky discoloration and delayed capillary refill of the left NAC with evolving epidermolysis and partial-thickness necrosis.

**Figure 3:**
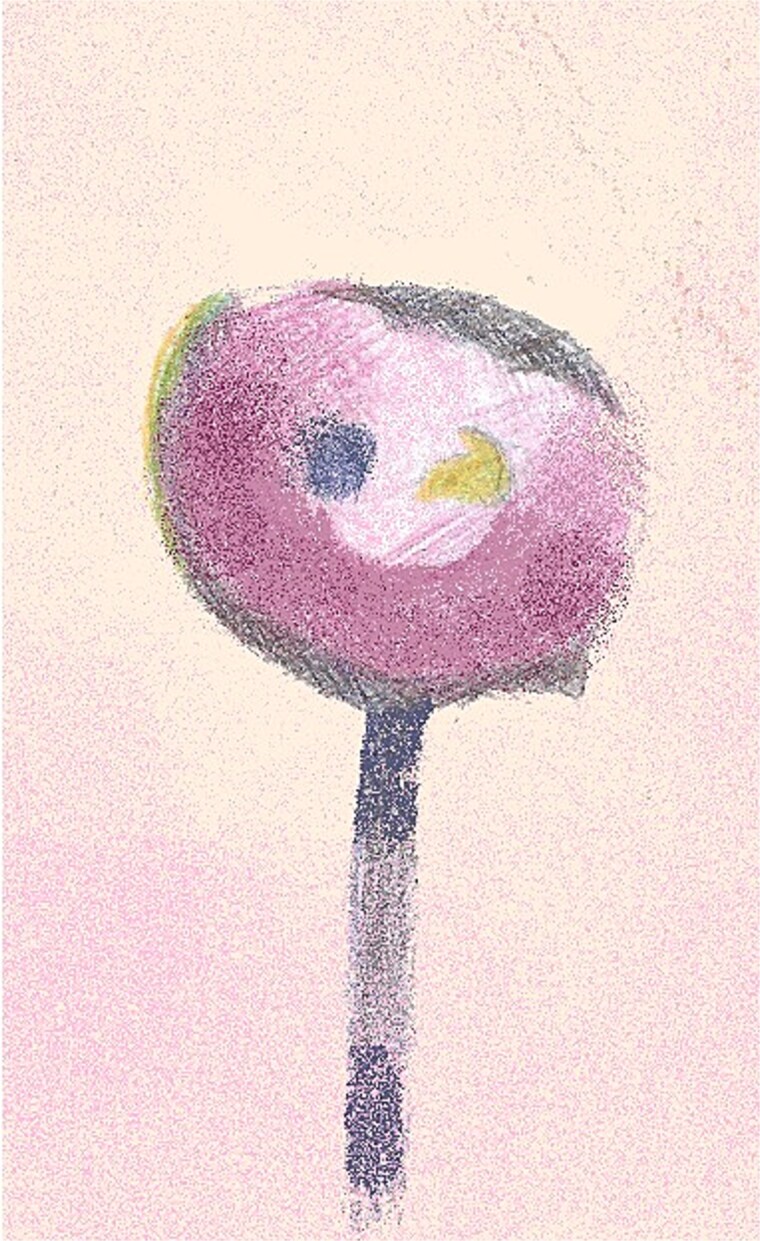
An illustration of left NAC necrosis as seen at Day 13 with adherent eschar and epidermolysis with central preservation of the nipple and surrounding areolar tissue.

**Figure 4:**
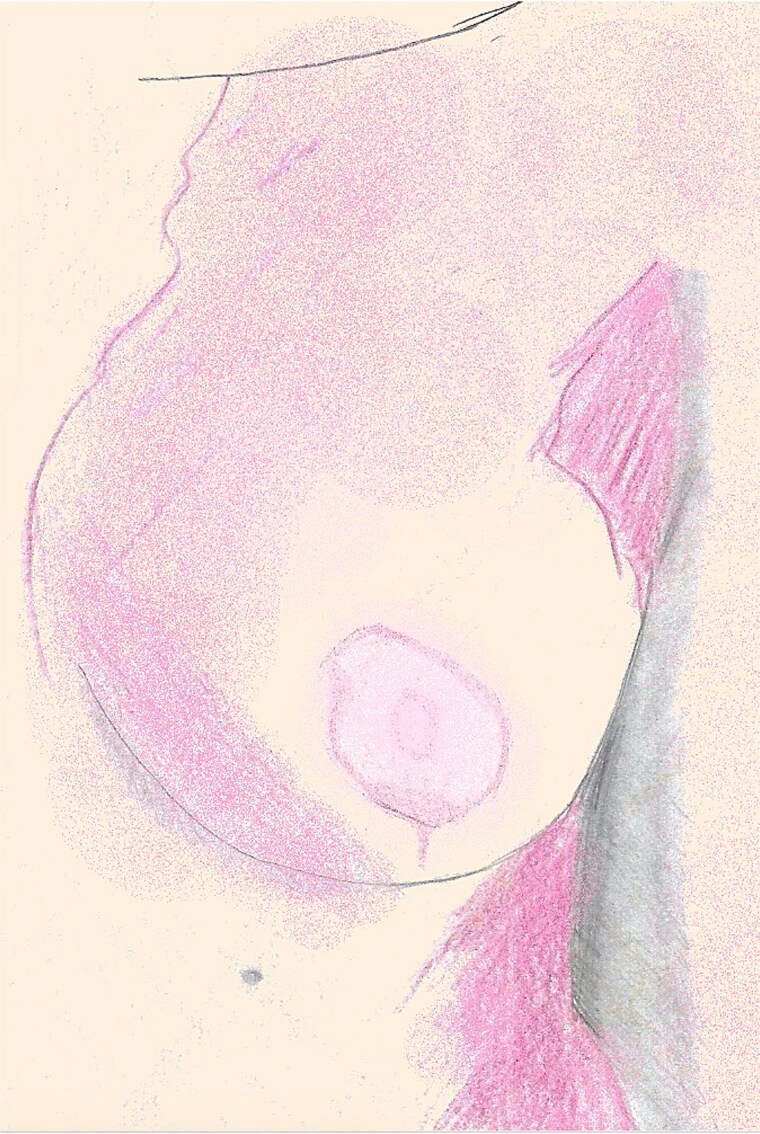
An illustration of the left breast 12 weeks postoperative, fully re-epithelialized with preservation of contour, projection, and minimal hypopigmentation.

Topical nitroglycerin ointment was administered to enhance perfusion but discontinued within 48 h due to intractable headaches. DMSO 2% solution was then applied topically to ischemic NAC three times daily. The patient reported progressive improvement in NAC color. The zone of necrosis remained limited to the peripheral areola, with the central nipple tissue remaining viable. On clinical examination at postoperative day 13, demarcation of areolar necrosis was evident along the peripheral left NAC from ~1 to 3 o’clock and 4 to 8 o’clock (Fig. 3). The affected regions showed adherent eschar and epidermolysis with central preservation of the nipple and surrounding areolar tissue, which remained pink and viable. By 8 weeks, ~90% of the areola had epithelialized with residual pink granulation tissue. At 12 weeks, the NAC was fully re-epithelialized with preservation of contour, projection, and minimal hypopigmentation (Fig. 4).

## Discussion

To our knowledge, this is the first reported case of NAC necrosis following mastopexy in a patient with KTS and prior pulsed dye laser therapy to the breast, successfully managed with topical DMSO. It highlights the interplay between underlying CVMs and iatrogenic vascular injury as contributors to compromised perfusion in reconstructive surgery.

Although the patient had known vascular malformation and prior PDL therapy, the etiology of NAC ischemia was likely multifactorial. We cannot definitively exclude the role of intraoperative factors such as pedicle dissection, mechanical tension, or flap handling. Intraoperative perfusion imaging was not performed, representing a limitation in assessing vascular integrity and potentially guiding intraoperative decision-making. Furthermore, while the necrosis remained partial thickness and localized, complications affecting the NAC are inherently significant in aesthetic breast surgery due to their potential long-term impact on appearance and patient satisfaction.

KTS involves abnormal embryologic vascular development. These lesions can impair nutrient delivery and reduce tissue resilience under stress [2, 3]. Prior reports have documented increased postoperative complications in KTS patients, particularly with large flaps or implants [2, 3].

PDL causes subclinical damage to blood vessels through repeated photothermal injury. Studies have shown decreased vessel density and signs of dermal sclerosis on histology years after treatment, raising concerns about its long-term effects on tissue perfusion [5, 6]. Huikeshoven *et al*. demonstrated that CVMS often re-darken due to vessel remodeling, suggesting incomplete or aberrant revascularization post-PDL [4]. Such alterations in tissue perfusion may have reduced capacity to accommodate surgical insult.

The therapeutic use of DMSO in this case is notable. In ischemic models, it has been shown to increase tissue survival, reduce necrosis, and enhance angiogenic signaling pathways [7–9]. In human studies, it has been effective in managing chemotherapy injuries and ischemic flaps [7, 9]. DMSO may improve perfusion in compromised tissue by reducing oxidative stress. In contrast, Nitroglycerin ointment improves perfusion via vasodilation but often causes side effects such as headache [10]. Overall, DMSO seems better tolerated. Its favorable safety profile and ease of topical application make it a promising alternative when nitroglycerin is not tolerated or insufficient.

Although a single case, the positive response to DMSO suggests potential benefit for other patients with microvascular compromise. Further studies are needed to define optimal concentration, timing, and duration of treatment. Additionally, future work should investigate preoperative vascular imaging or laser Doppler assessments in KTS- or PDL-treated patients undergoing breast surgery.

## Conclusion

Patients with KTS and prior PDL treatment of the breast present unique challenges during reconstructive surgery. They may be at risk for NAC necrosis even without traditional risk factors. Preoperative planning, early recognition of vascular insufficiency, and thoughtful use of pharmacologic adjuncts are essential in this complex patient population.
